# Regional data on electricity consumption and electricity prices in Japan

**DOI:** 10.1016/j.dib.2023.109467

**Published:** 2023-08-02

**Authors:** Akihiro Otsuka

**Affiliations:** Yokohama City University, Association of International Arts and Science, 22-2 Seto, Kanazawa, Yokohama 236-0027, Japan

**Keywords:** Residential electricity consumption, Industrial electricity consumption, Electricity consumption intensity, Electricity price, Price sensitivity

## Abstract

This dataset's data relate to Japan's electricity consumption from 1990 to 2015. It can be used by researchers, industry practitioners, and policymakers to analyze the impact of changes in electricity prices on the demand for electricity by region in the residential and industrial sectors. The dataset is divided into three categories: (i) regional sectoral electricity consumption data, (ii) regional sectoral electricity consumption intensity data, and (iii) regional sectoral electricity price data. Each category was obtained on an annual basis directly from various government databases. All data are aggregated by region.

**Specifications Table**

**Table:** 

Subject	Economics, Econometrics, and Finance
Specific subject area	Electricity Consumption; Electricity Consumption Intensity; Electricity Prices
Type of data	Table, Excel file
How the data were acquired	Datasets were compiled from various publicly available sources, as follows: Electric Utility Handbook (Ministry of Economy, Trade and Industry), Annual Securities Report (Power Company), Economic Census (Ministry of Economy,
	Trade and Industry), Basic Resident Registers (Ministry of Internal Affairs and Communications), Consumer Price Index (Ministry of Internal Affairs and Communications), and Corporate Goods Price Index (Bank of Japan).
Data format	Raw data were submitted alongside the data article.
Description of data collection	These data were collected when electric power companies had regional monopolies. From the postwar period to 2015, each electric power company in Japan had a monopoly on electricity supply, by area of supply. With the complete liberalization of the electricity market in 2016, this regional monopoly was dissolved, and electric power companies could sell electricity beyond their supply area. The ability of power companies to offer different electricity prices for each contract made it difficult to compute region-specific data on electricity prices; therefore, the scope of the data is limited to 2015.
Data source location	Electric Utility Handbook (Ministry of Economy, Trade and Industry), Annual Securities Report (Power Company), Economic Census (Ministry of Economy, Trade and Industry), Basic Resident Registers (Ministry of Internal Affairs and Communications), Consumer Price Index (Ministry of Internal Affairs and Communications), and Corporate Goods Price Index (Bank of Japan)
Data accessibility	The data can be accessed at the following link: https://data.mendeley.com/datasets/bc3p4phpy5/1
Related research article	Akihiro Otsuka, Industrial electricity consumption efficiency and energy policy in Japan, Util. Pol. 81 (2023) 101519 [1]. https://doi.org/10.1016/j.jup.2023.101519

## Value of the Data

• Databases held by international organizations such as the Organization for Economic Co-operation and Development and the International Energy Agency contain extensive data on electricity consumption by country. However, data by region within a country are incredibly scarce, mainly data on regional electricity prices. Therefore, the data on electricity consumption and electricity prices by region included in this dataset are helpful for the historical analysis of electricity demand by region and comparison of electricity consumption patterns by region.
• This dataset allows us to estimate the regional price sensitivity of electricity demand in Japan's residential and industrial sectors.
• Data on electricity prices, filtered by region, can be used to test various hypotheses about electricity price changes that interest scientists, the electricity industry, and energy policymakers.
• Researchers and data analysts can use these data for statistical and econometric modeling to characterize the relationship between energy prices and energy consumption in the residential and industrial sectors to provide policy implications.

## 1 Objective

The Great East Japan Earthquake occurred in Tohoku in 2011, resulting in a significant change in the national energy policy. First, the earthquake halted nuclear power generation and replaced it with thermal power generation. As a result, the electricity supply was severely constrained and energy conservation was promoted nationwide. In addition, the Japanese government revised the Basic Energy Plan to address global warming and aimed to build an electricity supply system centered on renewable energy sources. This led to an increase in the unit cost of electricity generation, which in turn caused electricity prices to rise nationwide. This dataset was created to examine the impact of electricity price fluctuations associated with these policy changes on regional electricity consumption.

## 2 Data Description

The data presented in this paper contain eight variables and are collated in a single Excel file. The file can be accessed at the following link: https://data.mendeley.com/datasets/bc3p4phpy5/1 [2].

Table 1 describes all variables. The data contain information on regional electricity consumption, intensity, and prices.

**Table 1:** Description of variables in the dataset.

Variable name	Description
**Electricity consumption per sector**	
Con. Res.	Total electricity consumption in the residential sector (kWh)
Con. Ind.	Total electricity consumption in the industry sector (kWh)
**Electricity consumption intensity per sector**	
EI. Res.	Electricity consumption per household in the residential sector (kWh)
EI. Ind.	Electricity consumption per business establishment in the industry sector (kWh)
**Electricity price per sector**	
N. Price. Res.	Nominal electricity prices in the residential sector (yen/kWh)
N. Price. Ind.	Nominal electricity prices in the industry sector (yen/kWh)
R. Price. Res.	Real electricity prices in the residential sector (2010=100)
R. Price. Ind.	Real electricity prices in the industry sector (2010=100)

The regional classification is as follows: Hokkaido (Hokkaido), Tohoku (Aomori, Iwate, Miyagi, Akita, Yamagata, Fukushima, and Niigata), Tokyo (Saitama, Chiba, Tokyo, Kanagawa, Ibaraki, Tochigi, Gunma, and Yamanashi), Hokuriku (Toyama, Ishikawa, and Fukui), Chubu (Nagano, Gifu, Shizuoka, Aichi, and Mie), Kansai (Shiga, Kyoto, Osaka, Hyogo, Nara, and Wakayama), Chugoku (Tottori, Shimane, Okayama, Hiroshima, and Yamaguchi), Shikoku (Tokushima, Kagawa, Ehime, and Kochi), Kyushu (Fukuoka, Saga, Nagasaki, Kumamoto, Oita, Miyazaki, and Kagoshima), and Okinawa (Okinawa).

Table 2 presents the descriptive statistics of electricity consumption by region. Tokyo, where the capital is located, has the highest electricity consumption in the residential and industrial sectors, while Okinawa, a remote island, has the lowest. Regarding the scale of demand, industrial electricity demand greatly exceeds residential electricity demand in all regions. Compared to residential electricity, the coefficient of variation for industrial electricity is small, and the annual fluctuations are negligible. Hokuriku has the most significant coefficient of variation in the residential sector and Okinawa has the most significant coefficient in the industrial sector.

**Table 2:** Descriptive statistics of regional electricity consumption (kWh).

	Residential sector			Industry sector		
	Mean	Standard deviation	CV	Mean	Standard deviation	CV
Hokkaido	10,474,488,692	1,504,082,176	14.36	17,648,982,154	2,157,440,711	12.22
Tohoku	22,001,093,000	3,169,745,564	14.41	49,808,460,846	5,643,444,518	11.33
Tokyo	85,184,314,808	11,498,150,400	13.50	181,034,024,269	12,434,451,269	6.87
Chubu	31,348,282,077	4,445,712,197	14.18	89,060,958,346	5,588,568,903	6.27
Hokuriku	6,854,567,769	1,289,171,982	18.81	18,918,719,231	1,298,936,692	6.87
Kansai	43,902,761,962	5,513,741,165	12.56	94,217,047,077	4,163,705,252	4.42
Chugoku	16,341,284,846	2,497,401,261	15.28	38,224,591,231	3,374,718,233	8.83
Shikoku	8,538,787,038	1,144,102,922	13.40	16,857,755,731	1,693,718,164	10.05
Kyushu	25,453,418,423	4,072,640,147	16.00	49,824,683,769	5,739,283,800	11.52
Okinawa	2,611,586,308	362,817,765	13.89	4,082,426,615	527,576,301	12.92

Note: See the main text for the regional classification.

Table 3 illustrates the descriptive statistics of electricity consumption intensity by region. The highest electricity consumption intensity for both the residential and industrial sectors is in Hokuriku. Regarding the scale of electricity consumption intensity, industrial electricity greatly exceeds residential electricity consumption in all regions. Compared to industrial electricity, the coefficient of variation for residential electricity consumption is small, and annual fluctuations are negligible. Hokuriku has the most significant coefficient of variation for the residential sector, while Hokkaido has the most significant for the industrial sector.

**Table 3:** Descriptive statistics of regional electricity consumption intensity (kWh).

	Residential sector			Industry sector		
	Mean	Standard deviation	CV	Mean	Standard deviation	CV
Hokkaido	4245	348	8.19	66,515	11,581	17.41
Tohoku	5303	468	8.82	79,858	13,381	16.76
Tokyo	5072	309	6.09	95,348	8523	8.94
Chubu	5195	373	7.18	100,026	11,113	11.11
Hokuriku	6620	788	11.90	102,370	14,276	13.94
Kansai	5398	347	6.43	90,816	8596	9.47
Chugoku	5511	529	9.61	98,862	14,341	14.51
Shikoku	5284	440	8.33	76,343	13,026	17.06
Kyushu	4855	456	9.39	76,555	12,321	16.09
Okinawa	5362	316	5.89	56,693	8520	15.03

Note: See the main text for the regional classification.

Table 4 presents the descriptive statistics of nominal electricity prices by region. The electricity price level is higher in the residential sector than in the industrial one. Hokkaido has the most significant coefficient of variation for both the residential and industrial sectors. Compared to residential electricity, the coefficient of variation for industrial electricity is smaller and annual fluctuations are minor.

**Table 4:** Descriptive statistics of nominal electricity prices (yen/kWh).

	Residential sector			Industry sector		
	Mean	Standard deviation	CV	Mean	Standard deviation	CV
Hokkaido	23.72	2.64	11.13	16.49	2.46	14.93
Tohoku	23.56	1.92	8.14	16.00	1.84	11.50
Tokyo	23.59	1.85	7.83	16.16	1.90	11.75
Chubu	22.65	1.28	5.63	15.65	1.30	8.33
Hokuriku	21.51	2.32	10.80	14.32	1.44	10.03
Kansai	22.24	1.63	7.32	15.35	1.48	9.63
Chugoku	22.83	2.05	9.00	14.93	1.56	10.46
Shikoku	22.79	1.89	8.30	15.92	1.93	12.14
Kyushu	22.39	2.40	10.71	15.69	1.98	12.59
Okinawa	23.91	1.00	4.19	18.63	1.13	6.04

Note: See the main text for the regional classification.

Table 5 illustrates the descriptive statistics of the real electricity prices by region. There are no significant differences in the real electricity price levels among sectors. The coefficient of variation of real electricity prices is the largest in Hokkaido for both sectors. There is no significant difference in the level of the coefficient of variation between sectors.

**Table 5:** Descriptive statistics of real electricity prices (Y2010=100).

	Residential sector			Industry sector		
	Mean	Standard deviation	CV	Mean	Standard deviation	CV
Hokkaido	113.69	13.34	11.74	117.02	14.17	12.11
Tohoku	112.19	9.47	8.44	113.28	10.01	8.83
Tokyo	111.34	8.70	7.81	115.18	11.29	9.80
Chubu	107.30	6.00	5.59	110.01	7.27	6.61
Hokuriku	116.20	13.15	11.32	112.64	8.81	7.82
Kansai	111.87	8.19	7.32	113.50	9.44	8.32
Chugoku	111.94	10.77	9.62	112.86	8.69	7.70
Shikoku	111.77	9.84	8.80	115.15	11.03	9.58
Kyushu	116.68	12.98	11.12	118.61	12.27	10.34
Okinawa	101.32	4.78	4.72	104.33	3.50	3.35

Note: See the main text for the regional classification.

## 3 Experimental Design, Materials and Methods

### 3.1 Electricity Consumption

The electricity consumption data for each sector can be obtained from the Electricity Demand Data in the Electricity Utilities Handbook. The electricity consumption in the residential sector is the total electricity consumed by each region in the residential sector, while the electricity consumption in the industrial sector is the total electricity consumed by each region in the industrial sector.

### 3.2 Electricity Consumption Intensity

The electricity consumption intensity data for each sector comprise electricity consumption data processed to identify specific consumption patterns. The electricity consumption intensity of the residential sector is the consumption unit, that is, the amount of electricity consumed per household. These data are calculated by dividing the total electricity consumption of the region by the number of households listed in the “basic resident register.”

The electricity consumption intensity of the industrial sector is the electricity consumption per unit of consumption, that is, per business establishment. These data are calculated by dividing a region's total electricity consumption by the number of establishments listed in the Economic Census.

### 3.3 Electricity Prices

The electricity prices for each sector are computed according to the consumption patterns in each sector.

The nominal electricity price is the electricity price per unit of consumption in each sector. It is obtained by dividing the total electricity sales of the electric power companies listed in the annual securities report by the total electricity consumption in each region.

Real electricity rates are calculated differently for the residential and industrial sectors. The real electricity rates for the residential sector are obtained by deflating the nominal unit price of the residential sector in the region by the “consumer price index.” The real electricity rates for the industrial sector are obtained by deflating nominal unit prices for the industrial sector in the region by the “corporate goods price index.”

## Ethics Statement

This dataset does not include human or animal-testing data. Permission is not required to use the primary data in this dataset, as it is free of charge.

## Declaration of Competing Interest

The author declares that he has no known competing financial interests or personal relationships that could have appeared to influence the work reported in this paper.

## Data Availability

regional-data-on-the-electricity-consumption-in-Japan (Original data) (Mendeley Data).
